# Increased Epithelial Oxygenation Links Colitis to an Expansion of Tumorigenic Bacteria

**DOI:** 10.1128/mBio.02244-19

**Published:** 2019-10-01

**Authors:** Stephanie A. Cevallos, Jee-Yon Lee, Connor R. Tiffany, Austin J. Byndloss, Luana Johnston, Mariana X. Byndloss, Andreas J. Bäumler

**Affiliations:** a Department of Medical Microbiology and Immunology, School of Medicine, University of California at Davis, Davis, California, USA; Weill Cornell Medical College; Max Planck Institute, Berlin; Universite Paris Descartes Faculte de medecine Necker / INSERM U1002

**Keywords:** *Escherichia coli*, colibactin, colorectal cancer, microbiome

## Abstract

Intestinal inflammation is a risk factor for colorectal cancer formation, but the underlying mechanisms remain unknown. Here, we investigated whether colitis alters the colonic microbiota to enhance its cancer-inducing activity. Colitis increased epithelial oxygenation in the colon of mice and drove an expansion of Escherichia coli within the gut-associated microbial community through aerobic respiration. An aerobic expansion of colibactin-producing E. coli was required for the cancer-inducing activity of this pathobiont in a mouse model of colitis-associated colorectal cancer formation. We conclude that increased epithelial oxygenation in the colon is associated with an expansion of a prooncogenic driver species, thereby increasing the cancer-inducing activity of the microbiota.

## Abstract (precis)

One of the environmental factors important for colorectal cancer formation is the gut microbiota, but the habitat filters that control its cancer-inducing activity remain unknown. Here, we show that chemically induced colitis elevates epithelial oxygenation in the colon, thereby driving an expansion of colibactin-producing Escherichia coli, a prooncogenic driver species. These data suggest that elevated epithelial oxygenation is a potential risk factor for colorectal cancer formation because the consequent changes in the gut habitat escalate the cancer-inducing activity of the microbiota.

## INTRODUCTION

Colorectal cancer is a major cause of cancer-related death, but only about 20% of cases can be genetically attributed to familial history (1), suggesting that environmental factors play an important role in promoting tumorigenesis. One prime candidate for environmental factors is an altered colonic microbiota (2). Consistent with this idea, tumorigenic bacteria, such as colibactin-producing Escherichia coli, are found in significantly higher percentages in patients with colorectal cancer than in healthy controls (3). Benign polyps developing early in life of patients with familial adenomatous polyposis are covered by patchy bacterial biofilms containing colibactin-producing E. coli (4), suggesting a link between early neoplasia of the colon and tumorigenic bacteria.

One of the risk factors for developing colorectal cancer is inflammatory bowel disease (IBD), such as Crohn’s disease or ulcerative colitis, which affects 0.4% of the population in Europe and North America (5, 6). Ulcerative colitis increases the cumulative risk of colorectal cancer by 18% after 30 years of active disease (7). IBD is associated with dysbiosis in the gut microbiota, characterized by an elevated abundance of facultative anaerobic *Enterobacteriaceae* (8–10), a family that includes colibactin-producing E. coli of phylogroup B2 (11). Colitis can promote tumorigenesis in a mouse model by altering the microbiota composition and inducing an expansion of microorganisms with genotoxic capabilities, such a colibactin-producing E. coli (3). However, the mechanisms by which colitis or neoplasia drives an expansion of tumorigenic bacteria remain unknown. Here, we investigated how colitis promotes an expansion of colibactin-producing E. coli to identify potential risk factors for developing colorectal cancer.

## RESULTS

### Colitis increases epithelial oxygenation in the colon.

**FIG 1:**
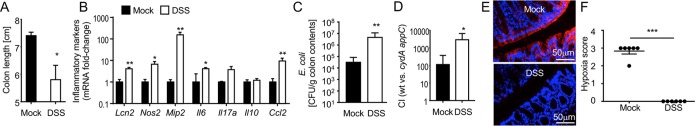
DSS-induced colitis increases epithelial oxygenation. Mice receiving 3% dextran sulfate sodium (DSS) in their drinking water were inoculated with commensal E. coli indicator strains (i.e., with a 1:1 mixture of E. coli Nissle 1917 wild type and an isogenic *cydA appC* mutant) 2 days after the beginning of DSS treatment. Organs were collected 4 days after the beginning of DSS treatment. (A) Colon length was determined at necropsy. (B) Transcript levels of the indicated inflammatory genes were determined in RNA isolated from the colonic epithelium using quantitative real-time PCR. (C) Numbers of E. coli bacteria recovered from colon contents. (D) The fitness advantage conferred by aerobic respiration was assessed by determining the competitive index (CI) of E. coli wild type (wt) and a *cydA appC* mutant recovered from colon contents. (E and F) Binding of pimonidazole was detected using Hypoxyprobe-1 primary antibody and a Cy3-conjugated goat anti-mouse secondary antibody (red fluorescence) in sections of the colon that were counterstained with DAPI nuclear stain (blue fluorescence). representative images are shown (E). Blinded sections of the colon were scored for intensity of hypoxia staining. Each dot represents data from one animal (F). For data in panels A to D, bars represent geometric means ± standard errors. *, *P < *0.05; **, *P < *0.01; ***, *P < *0.001.

Mice used in this study were obtained from the Jackson Laboratories because animals from this vendor do not carry endogenous *Enterobacteriaceae* (12), thus offering experimental control over the presence of E. coli. To investigate how mucosal inflammation alters the ecosystem in the gut lumen, we induced colitis in mice by providing dextran sulfate sodium (DSS) in the drinking water. DSS treatment triggered colitis, as indicated by a reduction in colon length (Fig. 1A) and increased expression of proinflammatory genes in the colonic mucosa (Fig. 1B). Inoculation of DSS-treated mice with a commensal E. coli isolate of phylogroup B2 (E. coli Nissle 1917 wild type) revealed that colitis drove a marked expansion of this facultative anaerobic bacterium (Fig. 1C), thus modeling the expansion of *Enterobacteriaceae* observed in patients with IBD (9). Since limited oxygen availability is thought to maintain a microbial community dominated by obligate anaerobic bacteria in the colon (13), we investigated whether an expansion of facultative anaerobic E. coli was linked to a disruption in anaerobiosis (8). Oxygen is readily consumed by facultative anaerobic bacteria in the gut lumen, which impedes direct oxygen measurements. We thus assessed the bioavailability of oxygen using bacterial indicator strains that were either aerobic respiration proficient (E. coli Nissle 1917 wild type) or deficient for aerobic respiration under microaerophilic conditions due to genetic ablation of cytochrome *bd* oxidase and cytochrome *bd-II* oxidase (E. coli Nissle 1917 *cydA appC* mutant). We then compared the fitness levels of the aerobic respiration-proficient and aerobic respiration-deficient bacteria by inoculating mice with a 1:1 mixture of both bioindicator strains. The fitness advantage conferred by aerobic respiration was significantly larger in DSS-treated mice than in control animals (Fig. 1D), which supported the idea that DSS treatment increases the bioavailability of oxygen in the intestinal lumen. Similar results were obtained when the experiment was repeated at a later time point after the beginning of DSS treatment (see Fig. S1A, S1B, S1C, and S1D in the supplemental material), when marked inflammatory changes were observed by histopathological scoring of sections from the colon (Fig. S1E). Recent work suggests that anaerobiosis in the colon is maintained by colonic epithelial cells (colonocytes), which consume oxygen through oxidative phosphorylation in their mitochondria, thereby maintaining the epithelial surface in a state of physiological hypoxia (14). To detect hypoxia, mice were injected with pimonidazole 1 h before euthanasia. Pimonidazole is reduced under hypoxic conditions to hydroxylamine intermediates that irreversibly bind to nucleophilic groups in proteins or DNA and can be detected by an antibody (15, 16). Pimonidazole staining revealed that the colonic surface of mock-treated mice was hypoxic. Notably, DSS-induced colitis resulted in a loss of epithelial hypoxia (Fig. 1E and F), suggesting that colitis was associated with increased epithelial oxygenation.

10.1128/mBio.02244-19.1 FIG S1 DSS-induced colitis drives a dysbiotic E. coli expansion through aerobic respiration. Mice receiving 3% DSS in their drinking water were inoculated with commensal E. coli indicator strains (i.e., with a 1:1 mixture of E. coli Nissle 1817 wild type and an isogenic *cydA appA* mutant) 4 days after the beginning of DSS treatment. Organs were collected 6 days after the beginning of DSS treatment. (A) Colon length was determined at necropsy. (B) Transcript levels of the indicated inflammatory genes were determined in RNA isolated from the colonic epithelium using quantitative real-time PCR. (C) Numbers of E. coli bacteria recovered from colon contents. (D) The fitness advantage conferred by aerobic respiration was assessed by determining the competitive index (CI) of E. coli wild type and *cydA appA* mutant recovered from colon contents. (E) Histopathological scoring of blinded colonic sections was performed by a veterinary pathologist. The histopathological score for each animal (bars) is shown. (F) Transcript levels of the indicated ER stress markers were determined in RNA isolated from the colonic epithelium using quantitative real-time PCR. (A to E) Bars represent geometric means ± standard errors. *, *P < *0.05; **, *P < *0.01; ***, *P < *0.001; ****, *P < *0.0001. Download FIG S1, PDF file, 0.02 MB. Copyright © 2019 Cevallos et al. 2019 Cevallos et al. This content is distributed under the terms of the Creative Commons Attribution 4.0 International license.

### DSS-induced colitis promotes a dysbiotic E. coli expansion through aerobic respiration.

**FIG 2:**
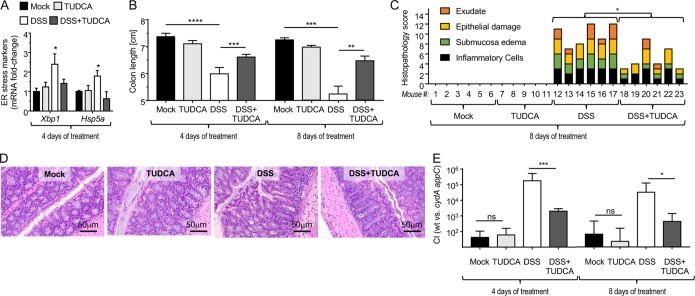
A DSS-induced increase in epithelial oxygenation promotes aerobic growth of E. coli. Mice received TUDCA daily by oral gavage. One day after the first TUDCA treatment, mice received drinking water containing 3% DSS. Two days after the beginning of DSS treatment, mice were inoculated with commensal E. coli indicator strains (i.e., with a 1:1 mixture of E. coli Nissle 1917 wild type and an isogenic *cydA appA* mutant). Organs were collected 4 or 8 days after the beginning of DSS treatment. (A) Transcript levels of the indicated UPR markers were determined in RNA isolated from the colonic epithelium using quantitative real-time PCR. (B) Colon length was determined at necropsy. (C and D) Histopathological scoring of blinded colonic sections was performed by a veterinary pathologist. The histopathological score for each animal (bar) is shown. Representative images of H&E-stained colonic sections are shown. (E) The fitness advantage conferred by aerobic respiration was assessed by determining the competitive index (CI) of E. coli wild type and a *cydA appA* mutant recovered from colon contents. For data in panels A, B, and E, bars represent geometric means ± standard errors. *, *P < *0.05; **, *P < *0.01; ***, *P < *0.001; ****, *P < *0.0001; ns, not statistically significantly different.

DSS treatment induces intestinal inflammation by triggering the unfolded protein response (UPR), thereby inducing endoplasmic reticulum (ER) stress in colonocytes (17, 18). Consistent with this idea, mRNA levels of genes encoding markers of ER stress were elevated in colonocytes from DSS-treated mice compared to levels in controls (Fig. S1F). Tauroursodeoxycholic acid (TUDCA) is a chemical chaperone that assists protein folding in the ER, thereby inhibiting induction of the UPR during DSS treatment (17, 18). To determine whether DSS treatment promoted aerobic growth of E. coli because it induced intestinal inflammation, we blocked intestinal inflammation during DSS treatment using TUDCA. TUDCA treatment blunted epithelial expression of genes encoding ER stress markers in DSS-treated mice (Fig. 2A) and reduced the severity of DSS-induced colitis, as indicated by a significant increase in colon length (Fig. 2B) and a reduced severity of inflammatory changes detected by histopathological analysis of sections from the colon (Fig. 2C and D). To determine whether reducing the severity of inflammation by TUDCA treatment was associated with diminished oxygen bioavailability, mice were inoculated with aerobic respiration-proficient (Nissle 1917 wild type) and aerobic respiration-deficient (Nissle 1917 *cydA appC* mutant) E. coli indicator strains. TUDCA treatment reduced the fitness advantage conferred by aerobic respiration in DSS-treated mice (Fig. 2E), which was indicative of reduced oxygen bioavailability. Collectively, these results suggested that DSS-induced intestinal inflammation drives a dysbiotic expansion of the commensal E. coli Nissle 1917 through aerobic respiration.

### AOM/DSS treatment drives an expansion of tumorigenic E. coli.

**FIG 3:**
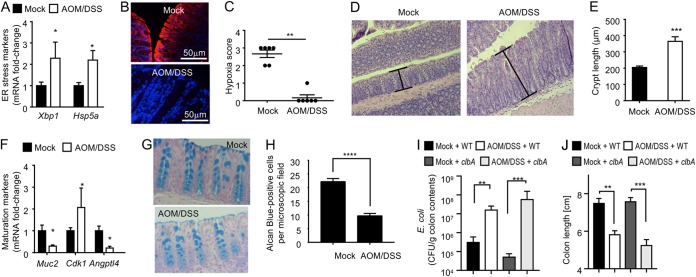
Short-term AOM/DSS treatment increases epithelial oxygenation. (A to G) Mice were treated with AOM, and 1 day later mice received 3% DSS in their drinking water for 1 week. Organs were collected 1 day after the end of DSS treatment. Transcript levels of the indicated UPR markers were determined in RNA isolated from the colonic epithelium using quantitative real-time PCR (A). (B and C) Binding of pimonidazole was detected using Hypoxyprobe-1 primary antibody and a Cy3-conjugated goat anti-mouse secondary antibody (red fluorescence) in sections of the colon that were counter stained with DAPI nuclear stain (blue fluorescence). Representative images are shown. Blinded sections of the colon were scored for intensity of hypoxia staining. Each dot represents data from one animal. (D and E) Crypt length was measured in H&E-stained histological sections from the colon. Representative images and a quantification of the average crypt lengths are shown. (F) Transcript levels of the indicated epithelial maturation markers were determined in RNA isolated from the colonic epithelium using quantitative real-time PCR. (G and H) Goblet cells were visualized histologically by alcian blue (mucin stain) staining, which is specific for sulfated and carboxylated acid mucopolysaccharides and sulfated and carboxylated sialomucins. Representative images and a quantification of the number of goblet cells are shown. (I and J) Mice were mock treated or treated with AOM and 1 day later inoculated with colibactin-producing E. coli strain SP15 (WT) or with a colibactin-deficient mutant (*clbA* strain). One day after inoculation with E. coli, AOM-treated mice received 3% DSS in their drinking water for 1 week. Organs were collected 1 day after the end of DSS treatment. Numbers of E. coli recovered from colon contents are indicated. (J) Colon length was determined at necropsy. *, *P < *0.05; **, *P < *0.01; ***, *P < *0.001; ****, *P < *0.0001.

Next, we wanted to investigate whether our results were relevant for the growth of tumorigenic E. coli in an animal model of colorectal cancer. The development of colitis-associated colorectal cancer can be modeled in DSS-treated mice that are pretreated with the genotoxic agent azoxymethane (AOM) (19). To determine whether an increase in epithelial oxygenation was also observed in the AOM/DSS model, mice were treated with AOM and then received DSS in their drinking water for 1 week (short-term AOM/DSS treatment). Similar to results in DSS-treated mice (Fig. S1E), transcript levels of genes encoding markers of ER stress were elevated in colonocytes after short-term AOM/DSS treatment (Fig. 3A). Pimonidazole staining revealed that short-term AOM/DSS treatment resulted in a loss of epithelial hypoxia (Fig. 3B and C), which was similar to the effect observed after treatment with DSS alone (Fig. 1E and F). Short-term AOM/DSS treatment resulted in colonic crypt hyperplasia, characterized by increased cell division leading to crypt elongation (Fig. 3D and E) and an elevated number of undifferentiated epithelial cells (Fig. 3F), as evidenced by increased epithelial transcript levels of *Cdk1*, a gene encoding a cyclin-dependent kinase involved in cell proliferation (20), and decreased epithelial transcript levels of *Angptl4*, a gene activated by (peroxisome proliferator-activated receptor gamma (PPARγ) in terminally differentiated epithelial cells (21, 22). Consistent with reduced epithelial differentiation, alcian blue staining revealed a reduction in the number of goblet cells, a terminally differentiated epithelial cell type, in colonic sections from AOM/DSS-treated mice compared to the level in control animals (Fig. 3G and H), which correlated with decreased epithelial transcript levels of *Muc2* (Fig. 3F). Importantly, short-term AOM/DSS treatment led to an expansion of colibactin-producing E. coli strain SP15 (3), which was independent of its ability to produce colibactin, because a similar expansion was observed in AOM/DSS-treated mice inoculated with an isogenic mutant deficient in colibactin production (*clbA* mutant) (Fig. 3I). Measurements of colon length (Fig. 3J) and histopathological analysis of sections from the colon (Fig. S2) suggested that colibactin production did not increase the severity of colitis during short-term AOM/DSS treatment.

10.1128/mBio.02244-19.2 FIG S2 Histopathology associated with short-term AOM/DSS treatment. Mice were treated with AOM and 1 day later mice received 3% DSS in their drinking water for 1 week. Organs were collected 1 day after the end of DSS treatment. Histopathological scoring of blinded colonic sections was performed by a veterinary pathologist. The histopathological score for each animal (bars) is shown. NS, not statistically significantly different. Download FIG S2, PDF file, 0.02 MB. Copyright © 2019 Cevallos et al. 2019 Cevallos et al. This content is distributed under the terms of the Creative Commons Attribution 4.0 International license.

### Expansion of tumorigenic E. coli through aerobic respiration enhances the cancer-inducing activity of the microbiota.

Having established that colitis promotes a dysbiotic E. coli expansion through aerobic respiration (Fig. 2E), we wanted to determine whether this mechanism could explain why intestinal inflammation increases the risk for colorectal cancer. Since inactivation of *cydA* and *appC* reduced the fitness of E. coli Nissle 1917 even in the absence of inflammation (Fig. 1D), we compared the fitness levels of the wild type and the *cydA appC* mutant during *in vitro* growth under microaerophilic conditions (0.8% O_2_) or anaerobic conditions. The *cydA appC* mutant exhibited reduced fitness during growth under microaerophilic conditions. This phenotype was reduced, but not abrogated, when the experiment was repeated under anaerobic conditions (Fig. S3A). A possible explanation for the residual fitness defect of a *cydA appC* mutant under anaerobic conditions is the role of cytochrome *bd-*II oxidase (encoded by *appC*) in detoxifying hydrogen peroxide, a metabolite that accumulates intracellularly when bacteria lack cytochrome *bd* oxidase (encoded by *cydA*) (23). We thus explored whether a *cydA* mutant would be a more specific bioindicator for oxygen availability. Notably, the *cydA* mutant had a fitness defect compared to growth of wild-type E. coli Nissle 1917 under microaerophilic conditions but not under anaerobic conditions (Fig. S3A). These data suggested that the growth defect of the *cydA* mutant was solely due to the presence of oxygen.

10.1128/mBio.02244-19.3 FIG S3 *In vitro* and *in vivo* growth of E. coli strains deficient for aerobic respiration under microaerophilic conditions. (A) Minimal medium containing glucose as a sole carbon source was inoculated with a 1:1 mixture of E. coli Nissle 1917 and either a *cydA appC* mutant or a *cydA* mutant. The competitive index was determined after a 24-h incubation at 37°C under anaerobic conditions or microaerophilic conditions. (B) E. coli requires aerobic respiration to reach high numbers in the feces during AOM/DSS-treatment. Mice were mock treated or treated with AOM and 1 day later inoculated with colibactin-producing E. coli strain SP15 (WT), with a colibactin-deficient mutant (*clbA* strain), with a cytochrome *bd* oxidase-deficient mutant (*cydA* strain), or with a mutant deficient for aerobic respiration under microaerophilic conditions (*cydA appC* strain). Mice received 3% DSS for 7 days starting at day 1, at 5 weeks, and at 10 weeks after E. coli inoculation. Bacterial shedding in the feces was monitored over time. Download FIG S3, PDF file, 0.03 MB. Copyright © 2019 Cevallos et al. 2019 Cevallos et al. This content is distributed under the terms of the Creative Commons Attribution 4.0 International license.

**FIG 4:**
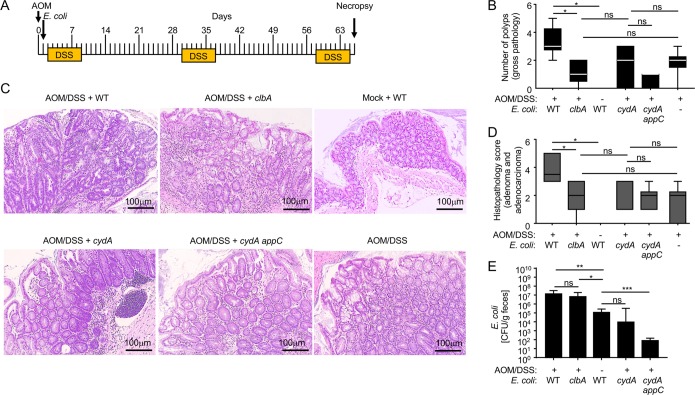
Expansion by aerobic respiration is required for the cancer-inducing activity of colibactin-producing E. coli. Mice were mock treated or treated with AOM and 1 day later inoculated with colibactin-producing E. coli strain SP15 (WT), with a colibactin-deficient mutant (*clbA* strain), with a cytochrome *bd* oxidase-deficient mutant (*cydA* strain), or with a mutant deficient for aerobic respiration under microaerophilic conditions (*cydA appC* strain). (A) Mice received courses of 3% DSS for 7 days starting at the indicated time intervals. (B) The number of polyps detected at necropsy by gross pathological inspection was recorded. The boxes in the whisker plot represent the first to third quartiles, and the mean value of the gross pathology scores is indicated by a line. Bars in the whisker plot represent the minimum and maximum data points within each treatment group. (C and D) The histopathology score was determined by a veterinary pathologist by scoring of blinded H&E-stained Swiss rolls of the colon. (C) Representative images of sections are shown. (D) The boxes in the whisker plot represent the first to third quartiles, and the mean value of the gross pathology scores is indicated by a line. Bars in the whisker pot represent the minimum and maximum data points within each treatment group. (E) E. coli numbers were determined in the colon contents collected during necropsy. Bars represent geometric means. Each dot represents data from one animal. *, *P < *0.05; **, *P < *0.01; ***, *P < *0.001; ns, not statistically significantly different.

Next, we determined whether an expansion of colibactin-producing E. coli by aerobic respiration was required for carcinogenesis during long-term AOM/DSS treatment (Fig. 4A). Consistent with a previous report (3), inoculation with colibactin-producing E. coli strain SP15 of phylogroup B2 led to the development of invasive mucinous adenocarcinoma after long-term AOM/DSS treatment. Increased disorganized hyperproliferation of colonic epithelium (hyperplasia/dysplasia), the presence of adenomatous polyp formation characterized by areas of proliferation of neoplastic colonic epithelial cells (i.e., epithelial cells that showed an increased nucleus/cytoplasm ratio), increased mitotic figures, and the presence of aberrant mitotic figures were observed in AOM/DSS-treated mice inoculated with colibactin-producing E. coli strain SP15 compared to levels in mice treated with AOM/DSS alone (Fig. 4B to D). The abundance of wild-type E. coli SP15 in the feces increased by 1 to 2 orders of magnitude following episodes of DSS treatment compared to levels in treatment-naive mice (Fig. S3B). Genetic ablation of colibactin production reduced polyp formation and the severity of neoplastic histopathologic lesions (Fig. 4A and B) without reducing the numbers of E. coli shed with the feces (Fig. S3B). These results confirmed that colibactin production is essential for carcinogenicity of E. coli SP15 (3). Genetic ablation of the ability to respire oxygen under microaerophilic conditions (*cydA appC* mutant) markedly reduced the ability of E. coli to colonize AOM/DSS-treated mice (Fig. 4C). An E. coli SP15 mutant that lacked only cytochrome *bd* oxidase (*cydA* mutant) showed enhanced intestinal colonization compared to that of a *cydA appC* mutant but was no longer able to reach high numbers in the feces during AOM/DSS treatment (Fig. S3B). At the end of the experiment (68 days after E. coli inoculation), the density of the *cydA* mutant in colon contents of AOM/DSS-treated mice was similar to that of the E. coli SP15 wild type in colon contents of mock-treated mice; however, the density of the E. coli SP15 wild type in colon contents of AOM/DSS-treated mice was approximately 100-fold higher (Fig. 4C). The inability of aerobic respiration-deficient E. coli strains (the *cydA* mutant and *cydA appC* mutant) to convert increased oxygen bioavailability into a growth advantage during AOM/DSS treatment abrogated their cancer-inducing activity because the severity of precancerous and neoplastic lesions was similar in AOM/DSS-treated mice that were mock inoculated or inoculated with an E. coli *cydA* mutant or an E. coli *cydA appC* mutant (Fig. 4A and B). Collectively, these data suggested that the ability to respire oxygen under microaerophilic conditions was essential for E. coli to expand in the intestine of AOM/DSS-treated mice (Fig. 4C) and that this dysbiotic expansion was required for the cancer-inducing activity of colibactin-producing E. coli (Fig. 4A and B).

## DISCUSSION

DSS-induced colitis in mice is a model that recapitulates relevant changes in epithelial metabolism observed in patients with ulcerative colitis. DSS treatment induces intestinal inflammation by triggering the unfolded protein response (UPR), thereby inducing endoplasmic reticulum (ER) stress in colonocytes (17, 18). The resulting epithelial injury induces excessive epithelial repair responses that lead to crypt elongation due to an accumulation of dividing transit-amplifying cells and a concomitant reduction in the number of terminally differentiated epithelial cells, such as goblet cells. A reduction in the number of terminally differentiated epithelial cells, such as goblet cells, and a concomitant thinning of the mucus layer are also observed in patients with ulcerative colitis (24, 25). Terminally differentiated epithelial cells obtain energy by degradation of fatty acids through β-oxidation and oxidative phosphorylation in the mitochondria, which results in high oxygen consumption (14, 26). In contrast, dividing transit-amplifying cells convert glucose into lactate even in the presence of oxygen, a metabolism characterized by low oxygen consumption (27). Consistent with an accumulation of dividing transit-amplifying cells, colonic epithelial cells from ulcerative colitis patients exhibit reduced mitochondrial β-oxidation of butyrate to carbon dioxide (28). This shift in epithelial metabolism is predicted to lower epithelial oxygen consumption. Importantly, ulcerative colitis is associated with an increased abundance of facultative anaerobic *Proteobacteria* in the colonic microbiota (29–31), which has been attributed to a disruption in anaerobiosis (8), but the mechanistic underpinnings have remained obscure. Here, we show that colitis was associated with increased epithelial oxygenation and increased oxygen bioavailability in the lumen, which drove an expansion of E. coli through growth by aerobic respiration. Thus, our finding that colitis increases epithelial oxygenation provides a mechanistic explanation for the dysbiotic expansion of facultative anaerobic *Proteobacteria* during chemically induced colitis.

Ulcerative colitis is a risk factor for developing colorectal cancer (7), but the underlying mechanism remains unknown. Colitis is associated with changes in the composition of the gut microbiota (29–31), and an altered colonic microbiota is thought to be one of the environmental factors that plays an important role in promoting colorectal cancer formation (2). E. coli bacteria are frequently found to colonize cancer lesions and neighboring epithelium, often accumulating there at large numbers, so that sometimes they are the only cultivable organisms in close contact to the diseased site (32–36). A higher fraction (42%) of E. coli isolates from colorectal cancer patients belong to phylogroup B2 than isolates from healthy controls (33). Colibactin-producing E. coli of phylogroup B2 escalates colorectal cancer formation in a mouse model of colitis-associated colorectal cancer formation (3), which makes this microbe a likely candidate for a prooncogenic driver species. Our work suggests that by increasing epithelial oxygenation, colitis drives an aerobic expansion of colibactin-producing E. coli, thereby increasing the cancer-inducing activity of the microbiota. Notably, an expansion of facultative anaerobic *Proteobacteria* in the colon is also observed for other conditions that are associated with an increased risk for colorectal cancer formation, including a high-fat diet (37, 38), antibiotic treatment (26), and alcohol dependence (39). In other words, many risk factors for colorectal cancer formation have in common that they generate an environment that favors an expansion of colibactin-producing E. coli. In the case of antibiotic treatment, the mechanism driving an expansion of E. coli is increased epithelial oxygenation in the colon (26), which we also observed in the DSS colitis model. Collectively, these data raise the possibility that increased epithelial oxygenation in the colon is a potential risk factor for colorectal cancer formation, a hypothesis that deserves further study.

## MATERIALS AND METHODS

### Contact for reagent and resource sharing.

Further information and requests for resources and reagents should be directed to and will be fulfilled by the lead contact, Andreas J. Bäumler (ajbaumler@ucdavis.edu).

### Experimental model and subject details. (i) Bacterial strains and culture conditions.

The E. coli strains were routinely grown aerobically at 37°C in LB broth (BD Biosciences) or on LB plates. When necessary, antibiotics were added to the medium at the following concentrations: 0.1 mg/ml carbenicillin, 0.05 mg/ml kanamycin, 0.03 mg/ml chloramphenicol, or 0.1 mg/ml streptomycin. When grown for competitive infection, strains were grown in a hypoxia chamber (0.8% oxygen) at 37°C in LB broth (BD Biosciences).

**(ii) Animal experiments.** All experiments in this study were approved by the Institutional Animal Care and Use Committee at the University of California at Davis. Female C57BL/6J wild-type mice, aged 8 to 10 weeks, were obtained from The Jackson Laboratory.

### (a) DSS treatment.

For short-term treatment, mice were given 3% DSS in their drinking water continuously for 8 days. At day 4 of DSS treatment, mice were inoculated with 1 × 10^9^ CFU of a 1:1 mixture of E. coli Nissle 1917 and the E. coli Nissle 1917 *cydA appC* mutant. Samples were collected at 4 days postinfection.

### (b) TUDCA treatment.

To collect data for 4 days of DSS treatment, mice were supplemented with 250 mg/kg/day of tauroursodeoxycholic acid (TUDCA; EDM Millipore) via oral gavage for 6 days. At day 2, mice were supplemented with 3% DSS continuously in the drinking water. At day 4, mice were inoculated with 1 × 10^9^ CFU of a 1:1 mixture of E. coli Nissle 1917 and the E. coli Nissle 1917 *cydA appC* mutant. Samples were collected at 2 days postinfection.

To collect data for 8 days of DSS treatment, mice were supplemented with 750 mg/kg/day of TUDCA via oral gavage for 9 days. At day 2, mice were supplemented with 3% DSS continuously in the drinking water. At day 6, mice were inoculated with 1 × 10^9^ CFU of a 1:1 mixture of E. coli Nissle 1917 and the E. coli Nissle 1917 *cydA appC* mutant. Samples were collected at 2 days postinfection.

### (c) AOM/DSS treatment.

Mice were treated with 10 mg/kg/animal of azoxymethane (AOM; Sigma) via intraperitoneal injection, followed by oral inoculation with 1 × 10^9^ CFU of E. coli strains at 24 h postinjection. Supplementation with 3% dextran sulfate sodium (DSS; Alfa Aesar) in the drinking water was continuously provided for a week postinfection. Two subsequent weeklong DSS supplementations were given in between 3-week rests during the 9-week experiment. The AOM/DSS short-term treatment samples were collected at day 8 of the first DSS treatment.

### Method details. (i) Colonocyte isolation.

The colon and part of cecum were opened lengthwise and cut into 2- to 4-cm pieces, collected in 15 ml of ice-cold 1× RPMI 1640 buffer (Gibco) in a 50-ml Falcon tube and cleaned with 20 ml of ice-cold 1× Dulbecco’s phosphate-buffered saline (DPBS; Gibco) in another 50-ml Falcon tube. The tissue was then placed into 15-ml conical centrifuge tubes filled with 10 ml of ice-cold dissociation reagent 1 (30 mM EDTA, 1.5 mM dithiothreitol [DTT], diluted into 1× DPBS) and placed on ice for 20 min. Tissues were then placed into a 15-ml conical centrifuge tube filled with 6 ml of warm (37°C) dissociation reagent 2 (30 mM EDTA, diluted into 1× DPBS) and incubated for 10 min at 37°C. After this incubation, tubes were shaken vigorously for 30 s to detach the epithelium from basement membrane, for a total of about 80 to 90 shake cycles. Remnant intestinal tissue was removed, and pellet cell solution was centrifuged at 800 × *g* for 5 min at 4°C. Supernatant was removed, and the cell pellet was resuspended in 1 ml of Tri reagent (Molecular Research Center) for subsequent RNA extraction.

**(ii) Real-time PCR.** RNA from colonocytes was isolated with Tri reagent (Molecular Research Center), according to the instructions of the manufacturer. mRNA was purified using an NEBNext poly(A) mRNA magnetic isolation module (New England BioLabs [NEB]) according to the instructions of the manufacturer. A reverse transcriptase reaction was performed to prepare complementary DNA (cDNA) using TaqMan reverse transcription reagents (Applied Biosystems). A volume of 4 μl of cDNA was used as the template for each real-time PCR in a total reaction volume of 25 μl. Real-time PCR was performed using SYBR green (Applied Biosystems) and the primers listed in Table S1 in the supplemental material. Data were analyzed using the comparative threshold cycle (*C_T_*) method (Applied Biosystems). Transcript levels of *Hsp5a*, *Xbp1*, *Nos2*, *Lcn2*, *Mip2*, *IL-6*, *Il17a*, *IL-10*, *Muc1*, *Muc2*, *Ccl2*, *Cdk1*, and *Angptl4* and were normalized to mRNA levels of the housekeeping gene *Act2b*, encoding β-actin.

10.1128/mBio.02244-19.4 TABLE S1 Primers for real-time PCR of murine genes. Download Table S1, PDF file, 0.02 MB. Copyright © 2019 Cevallos et al. 2019 Cevallos et al. This content is distributed under the terms of the Creative Commons Attribution 4.0 International license.

**(iii) Construction of E. coli mutants.** E. coli strain SP15 (40), E. coli strain Nissle 1917 (41), an E. coli strain SP15 *clbA* mutant (42) and an E. coli strain Nissle 1917 *cydA appC* mutant (YL17) (43) have been described previously. To construct an E. coli strain SP15 *cydA* mutant, upstream and downstream regions of approximately 0.5 kb in length flanking the E. coli *cydA* gene were amplified by PCR and purified using a MiniElute kit (Qiagen) using the primers listed in Table S2. The pRDH10 suicide vector was digested with SalI, run on an agarose gel, purified using a MiniElute kit (Qiagen), and assembled with the fragments using Gibson Assembly Master Mix (NEB) to form plasmid pSC1. Plasmid pSC1 was then transformed into E. coli SM10 λ*pir*, and conjugation was performed with E. coli strain SP15. Conjugation was performed at 37°C, and exconjugants in which the suicide plasmid had integrated into the chromosome were recovered on LB plates containing chloramphenicol and streptomycin. Subsequent sucrose selection was performed on sucrose plates (5% sucrose, 8 g/liter nutrient broth base, 15 g/liter agar) to select for the second crossover events. PCR was performed to detect events that led to the unmarked deletion of the *cydA* gene. An SP15 *cydA* mutant generated by this method was termed SC1. To create an *appC* mutant, upstream and downstream regions flanking the E. coli *appC* gene were amplified by PCR and introduced into the pRDH10 suicide vector to generate plasmid pSC1. Plasmid pSC1 was transformed into E. coli SM10 λ*pir* for conjugation into E. coli strain SP15 *cydA* (SC1). Conjugation was performed at 37°C, and exconjugants in which the suicide plasmid had integrated into the chromosome were recovered on LB plates containing chloramphenicol and streptomycin. Subsequent sucrose selection was performed on sucrose plates (5% sucrose, 8 g/liter nutrient broth base, 15 g/liter agar) to select for the second crossover events. PCR was performed to detect events that led to the unmarked deletion of the *cydA* and *appC* genes. An E. coli SP15 *cydA appC* mutant generated by this method was termed SC2.

10.1128/mBio.02244-19.5 TABLE S2 Primers for amplification of bacterial genes. Download Table S2, PDF file, 0.01 MB. Copyright © 2019 Cevallos et al. 2019 Cevallos et al. This content is distributed under the terms of the Creative Commons Attribution 4.0 International license.

**(iv) *In vitro* growth of E. coli Nissle 1917 strains under anaerobic or microaerophilic conditions.** Liquid overnight cultures of each E. coli strain were washed twice with phosphate-buffered saline (PBS), diluted to a concentration of 1 × 10^7^ CFU/ml, and mixed in a 1:1 ratio of the E. coli Nissle 1917 wild type and an isogenic mutant strain. These cultures were then diluted to a concentration of 1 × 10^6^ CFU/ml in M9 minimal medium (M9 salts medium with 0.1% glucose) and incubated statically for 24 h at 37°C in an anaerobic chamber (Shel Lab Bactron II; 5% hydrogen, 5% carbon dioxide, 90% nitrogen) or in a hypoxia chamber (Coy Laboratory Products) set at 0.8% oxygen. The competitive index (ratio of wild type/mutant) was determined by spreading serial 10-fold dilutions on LB agar plates containing the appropriate antibiotics.

**(v) Preparation of Swiss rolls.** Swiss rolls of the colon were prepared as described in more detail elsewhere (44). Briefly, the dissected colonic tissue was removed into a petri dish and then flushed with modified Bouin’s fixative (50% ethanol–5% acetic acid in distilled H_2_O [dH_2_O]) through an oral gavage needle. Once the fixative was flushed through the colon, it was then cut longitudinally and washed with buffered saline. The entire length of the colon was then unraveled, having the luminal side face up, and this was then rolled on a toothpick from the proximal colon to distal colon. The Swiss roll colon was then carefully transferred into a cassette in 10% formalin for later processing.

**(vi) Histopathological analysis.** Colon and cecal tissues were fixed in 10% phosphate-buffered formalin, and 5-μm sections of the tissue samples were stained with hematoxylin and eosin (H&E). Representative images were taken using an Olympus BX41 microscope at a magnification of ×10. Scoring of blinded tissue sections was performed by a veterinary pathologist based on the criteria listed in Table S3 for DSS treatment and Table S4 for AOM/DSS treatment.

10.1128/mBio.02244-19.6 TABLE S3 Criteria for scoring inflammatory changes in the intestinal mucosa. Download Table S3, PDF file, 0.01 MB. Copyright © 2019 Cevallos et al. 2019 Cevallos et al. This content is distributed under the terms of the Creative Commons Attribution 4.0 International license.

10.1128/mBio.02244-19.7 TABLE S4 Criteria for scoring malignant changes in the intestinal mucosa. Download Table S4, PDF file, 0.02 MB. Copyright © 2019 Cevallos et al. 2019 Cevallos et al. This content is distributed under the terms of the Creative Commons Attribution 4.0 International license.

**(vii) Measurement of colon crypt length.** Histopathology sections were blinded for quantification. Colon crypt length was measured by the distance from the basal toward the apical portion of the colonic crypt using ImageJ imaging software. On average, 10 crypts per mouse were measured. On the graphs, each bar represents the average per treatment group.

**(viii) Alcian blue staining.** Colon and cecal tissues were fixed in 10% phosphate-buffered formalin, and 5-μm sections of the tissue samples were stained with alcian blue staining. Pictures were captured on an Axiocam camera (2.2-μm by 2.2-μm pixel distance). Quantification of mature mucus-producing, alcian blue-positive cells was conducted based on a comet-like feature of the cells, with dense blue staining on the apical side of colonic crypt within the frame. Each measurement was conducted based on 5 frames per individual mouse; the average for each group was based on data from six mice per group determined at a magnification of ×40.

**(ix) Hypoxia staining.** For detection of hypoxia, mice were treated with 60 mg/kg of pimonidazole HCl intraperitoneally (i.p.) (Hypoxyprobe-1 kit; Hypoxyprobe) 1 h prior to euthanasia. Colon samples were fixed in 10% phosphate-buffered formalin, and paraffin-embedded tissue was blocked with mouse-on-mouse blocking reagent (Vector Labs) and probed with mouse anti-pimonidazole monoclonal IgG1 (monoclonal antibody [MAb] 4.3.11.3). Then, slides were stained with Cy3-conjugated goat anti-mouse antibody (Jackson ImmunoResearch Laboratories). Samples were counterstained with 4′,6′-diamidino-2-phenylindole (DAPI) using SlowFace Gold mountant. Samples were scored based on the degree of colonic epithelial hypoxia (0, no hypoxia; 1, mild focal hypoxia; 2, moderate multifocal hypoxia; 3, intense diffuse hypoxia). Representative images were obtained using a Zeiss Axiovert 200 M fluorescence microscope and were brightness adjusted.

**(x) Quantification and statistical analysis.** To determine fold changes of ratios (bacterial competitive index, mRNA levels, alcian blue quantification, colon crypt length measurement), bacterial numbers were transformed logarithmically prior to statistical analysis. To determine the statistical significance of differences in total histopathology scores, a Mann-Whitney *U* test was used. An unpaired Student's *t* test was used on the transformed data to determine whether differences between groups were statistically significant (*P* < 0.05).
