# Supplementary figures and images for: Increased Epithelial Oxygenation Links Colitis to an Expansion of Tumorigenic Bacteria
Source: mBio. 2019 Oct 1;10(5):e02244-19. doi: 10.1128/mBio.02244-19 (PMC6775460; doi:10.1128/mBio.02244-19)

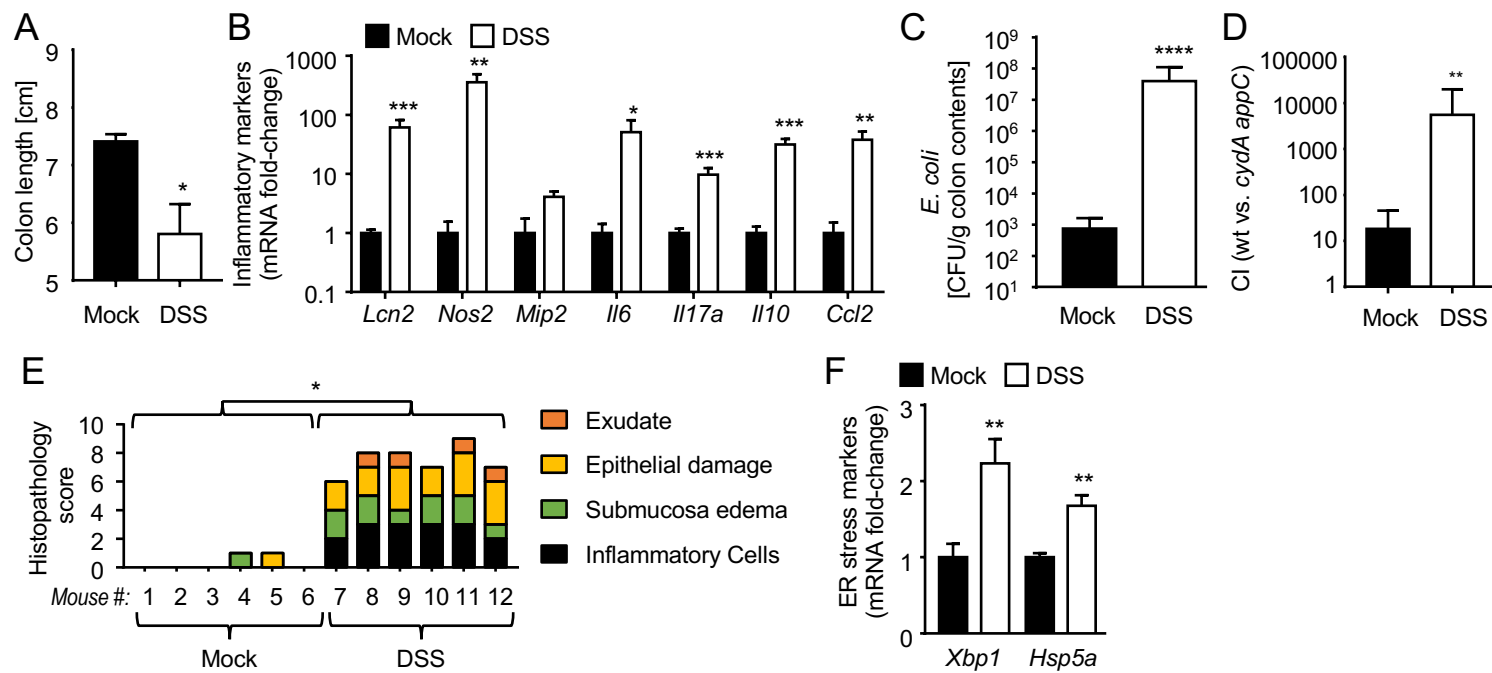

Figure S1

Supplement: FIG S1 [file mBio.02244-19-sf001.pdf]

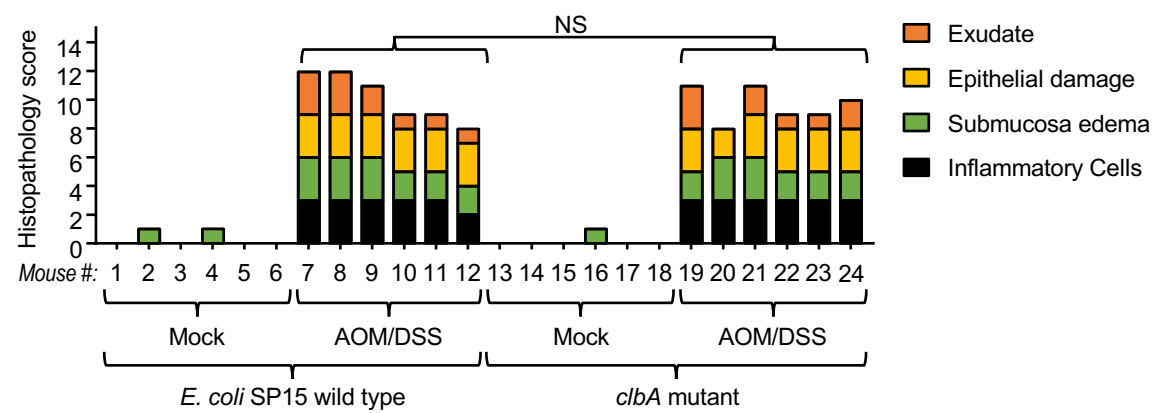

Figure S2

Supplement: FIG S2 [file mBio.02244-19-sf002.pdf]

A

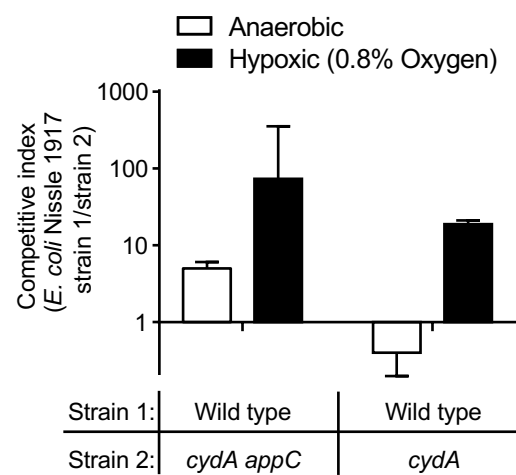

B

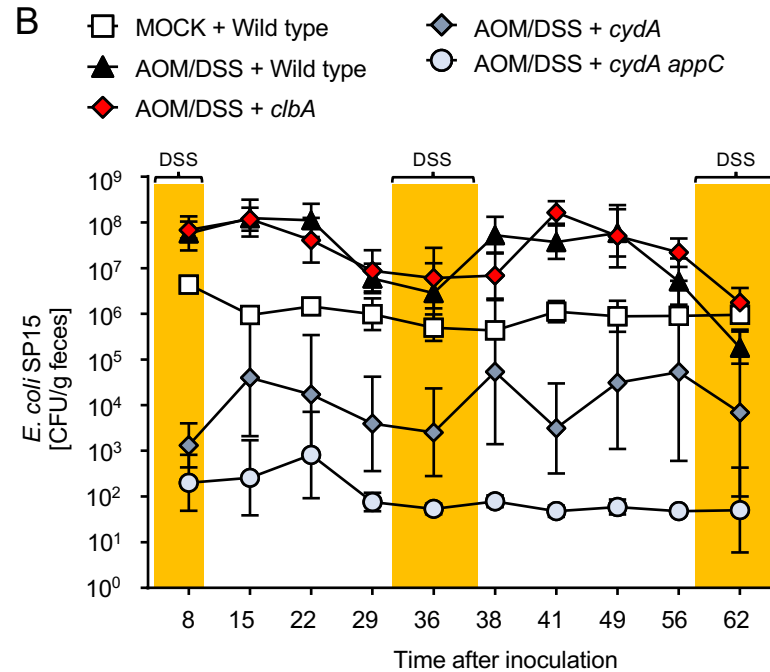

Figure S3

Supplement: FIG S3 [file mBio.02244-19-sf003.pdf]
