# Supplementary material for: Increased Epithelial Oxygenation Links Colitis to an Expansion of Tumorigenic Bacteria
Source: mBio. 2019 Oct 1;10(5):e02244-19. doi: 10.1128/mBio.02244-19 (PMC6775460; doi:10.1128/mBio.02244-19)
Supplement: TABLE S1 [file mBio.02244-19-st001.pdf]

Supplementary Table 1: Primers for real-time PCR of murine genes

GENE	FORWARD	REVERSE
Ccl2	5’-AGGTGTCCCAAAGAAGCTGTA-3’	5’-ATGTCTGGACCCATTCCTTCT-3’
Angptl4	5’-AGGCTGGACAGTTAATTCAGAGG-3’	5’-ATGCTATGCTATGCACCTTCTCCAGAC-3’
Il17a	5'-AACCCCCACGTTTCTCAGCAAAC-3'	5'-GGACCCCTTTACACCTTCTTTTCATTG-3'
Hsp5a	5'-GAGCGTCTGATTGGCGATGC-3'	5'-TTCCAAGTGCGTCCGATGAGG-3'
Lcn2	5'-ACATTTGTTCCAAGCTCCAGGGC-3'	5'-CATGGCGAACTGGTTGTAGTCCG-3'
Il10	5'-GGTTGCCAAGCCTTATCGGA-3'	5'-ACCTGCTCCACTGCCTTGCT-3'
Muc1	5'-GAAGACCCCAGCTCCAACTA-3'	5'-GGAGCCTGACCTGAACTTGA-3'
Muc2	5'-GTGTGGGACCTGACAATGTG-3'	5'-ACAACGAGGTAGGTGCCATC-3'
B-catenin	5'-ATGGAGCCGGACAGAAAAGC-3'	5'-TGGGAGGTGTCAACATCTTCTT-3'
Xbpi	5'-GAGTCCGCAGCAGGTG-3'	5'-GTGTCAGAGTCCATGGGA-3'
Ki67	5'-AGAAGTCCAGGTCTACAG-3'	5'-TCGTTGCTATTGCTAAGG-3'
Nos2	5'-TTGGGTCTTGTTCACTCCACGG-3'	5'-CCTCTTTCAGGTCACTTTGGTAGG-3'
Chop	5'-CTGGAAGCCTGGTATGAGGAT-3'	5'-CAGGGTCAAGAGTAGTGAAGGT-3'
Bactin	5'-TGTCCACCTTCCAGCAGATGT-3'	5'-AGCTCAGTAACAGTCCGCCTAGA-3'
Cdk1	5'-AGGTACTTACGGTGTGGTGTAT-3'	5'-CTCGCTTTCAAGTCTGATCTTCT-3'
