# Supplementary material for: Increased Epithelial Oxygenation Links Colitis to an Expansion of Tumorigenic Bacteria
Source: mBio. 2019 Oct 1;10(5):e02244-19. doi: 10.1128/mBio.02244-19 (PMC6775460; doi:10.1128/mBio.02244-19)
Supplement: TABLE S2 [file mBio.02244-19-st002.pdf]

Supplementary Table 2: Primers for amplification of bacterial genes

Purpose	FORWARD	REVERSE
Confirm deletion of *appC*	5'-CAGTTGCTGGAAGACAAACTGG-3'	5'-ATCACACCAACAGTTTTCAGT-3'
Confirm deletion of *cydA*	5'-AAATGGCAATAAAGTAGCTACTCT-3'	5'-ATACCGTACATCACCCATA-3'
Deletion of *cydA*: pRDH10 front BDI	5'-GCATAAGGGAGAGCGCAGATAAATTGAAGCAGTCTG-3'	5'-CTCCTGTCCATGACTCCTTGCTCATC-3'
Deletion of *cydA*: pRDH10 end BDI	5'-GAGTCATGGACAGGAGTCGTCAAATG-3'	5'-TCTCAAGGGCATCGGCCCACATTGTCAGACTTG-3'
