# Supplementary material for: Increased Epithelial Oxygenation Links Colitis to an Expansion of Tumorigenic Bacteria
Source: mBio. 2019 Oct 1;10(5):e02244-19. doi: 10.1128/mBio.02244-19 (PMC6775460; doi:10.1128/mBio.02244-19)
Supplement: TABLE S3 [file mBio.02244-19-st003.pdf]

Supplementary Table 3: Criteria for scoring inflammatory changes in the intestinal mucosa

Score	Infiltration inflammatory cells	Submucosal edema	Epithelial damage	Exudate
3	Severe multifocal to diffuse infiltration	Severe (>40%)	Multifocal to diffuse ulceration and/or severe multifocal to diffuse enterocyte hyperplasia with metaplasia	Severe accumulation
2	Moderate multifocal to diffuse infiltration	Moderate (20-40%)	Moderate multifocal to diffuse loss of goblet cells associated with enterocyte hyperplasia	Moderate accumulation
1	Mild multifocal diffuse infiltration	Mild (1-20%)	Erosion/mild loss of goblet cells and enterocyte hyperplasia	Mild accumulation
0	No infiltration	Absent	Absent	Absent
