# Supplementary material for: Increased Epithelial Oxygenation Links Colitis to an Expansion of Tumorigenic Bacteria
Source: mBio. 2019 Oct 1;10(5):e02244-19. doi: 10.1128/mBio.02244-19 (PMC6775460; doi:10.1128/mBio.02244-19)
Supplement: TABLE S4 [file mBio.02244-19-st004.pdf]

Supplementary Table 4: Criteria for scoring malignant changes in the intestinal mucosa

Score	Precancerous lesions	Cancerous lesions
0	No lesion	No lesion
1	Mild focal to multifocal hyperproliferation of colonic epithelium and/or presence of 1 adenomatous polyp (adenoma)	Presence of 1 malignant polyp (adenocarcinoma) characterized by proliferation of neoplastic epithelial cells associated with invasion through the muscularis mucosae into the submucosa
2	Moderate multifocal hyperproliferation of colonic epithelium and/or presence of 2-3 adenomatous polyp (adenoma)	Presence of 2-3 malignant polyps (adenocarcinoma) characterized by proliferation of neoplastic epithelial cells associated with invasion through the muscularis mucosae into the submucosa
3	Marked multifocal to diffuse hyperproliferation of colonic epithelium and/or presence of > 3 adenomatous polyp (adenoma)	Presence of >3 malignant polyps (adenocarcinoma) characterized by proliferation of neoplastic epithelial cells associated with invasion through the muscularis mucosae into the submucosa
